# Improved Trapping and Handling of an Arboreal, Montane Mammal: Red Panda *Ailurus fulgens*

**DOI:** 10.3390/ani11040921

**Published:** 2021-03-24

**Authors:** Damber Bista, Sonam Tashi Lama, Janno Weerman, Ang Phuri Sherpa, Purushotam Pandey, Madhuri Karki Thapa, Haribhadra Acharya, Nicholas J. Hudson, Greg S. Baxter, Peter John Murray

**Affiliations:** 1 School of Agriculture and Food Sciences, The University of Queensland, Gatton, QLD 4343, Australia; n.hudson@uq.edu.au (N.J.H.); gregbaxter36@gmail.com (G.S.B.); 2 Red Panda Network, Baluwatar, Kathmandu 44600, Nepal; sonam.lama@redpandanetwork.org (S.T.L.); ang.sherpa@redpandanetwork.org (A.P.S.); 3 Rotterdam Zoo, Blijdorplaan 8, 3041 JG Rotterdam, The Netherlands; j.weerman@diergaardeblijdorp.nl; 4 Directorate of Livestock and Fisheries Development, Province no.1, Biratnagar 56613, Nepal; wildelephant.pandey@gmail.com; 5 Department of Forest and Soil Conservation, Babarmahal, Kathmandu 44600, Nepal; madhureethapa@gmail.com; 6 Department of National Parks and Wildlife Conservation, Babarmahal, Kathmandu 44600, Nepal; hbacharya07@gmail.com; 7 School of Sciences, University of Southern Queensland, West St, Darling Heights, QLD 4350, Australia; peter.murray2@usq.edu.au

**Keywords:** animal handling, arboreal mammal, cage-trap, fencing-method, GPS collaring, immobilization, morphometric data, red panda trapping

## Abstract

**Simple Summary:**

Capture and handling is essential to study some biological and ecological properties of free-ranging animals. However, capturing an arboreal and cryptic species such as the red panda is challenging due to the difficult terrain, their elusive nature, and potential risks to human and animal safety. We developed and successfully tested a protocol for tracking, capture, immobilization, and handling of red pandas. This method could also be used, with some modifications, for other arboreal species. This study extends the known range of body weight and length of free-ranging red pandas. We also report some new morphometric data that could serve as a guide for field identification.

**Abstract:**

It is sometimes essential to have an animal in the hand to study some of their ecological and biological characteristics. However, capturing a solitary, cryptic, elusive arboreal species such as the red panda in the wild is challenging. We developed and successfully tested a protocol for tracking, trapping, immobilization, and handling of red pandas in the wild in eastern Nepal. We established a red panda sighting rate of 0.89 panda/day with a capture success rate of 0.6. We trapped and collared one animal in 3.7 days. On average, we took nearly 136 (range 50–317) min to capture an animal after spotting it. Further processing was completed in 38.5 (21–70) min. Before capture, we found it difficult to recognize the sex of the red panda and to differentiate sub-adults above six months from adults. However, body weight, body length, tail length, shoulder height, and chest girth can be used for diagnosis, as these attributes are smaller in sub-adults. Our method is a welfare-friendly way of trapping and handling wild red pandas. We report new morphometric data that could serve as a guide for field identification.

## 1. Introduction

Capture and handling of live animals is essential to study aspects of their biology and ecology in the field. Successful trapping and handling of study animals may determine the success of such studies. However, improper capture methods, such as leg snares, can have long-term adverse effects on study animals [1]. Hence, the welfare of study animals and human safety are major concerns in animal capture and handling [2,3]. In this regard, capturing a solitary free-ranging arboreal mammal in a remote, montane landscape such as the temperate Himalayan forests is challenging. In many, if not most, wildlife studies workers must also cope within the constraints of difficult access, time pressure and limited finances. We describe an effective, welfare-friendly protocol developed for capturing and handling the arboreal red panda (*Ailurus fulgens*), adapted from previous work on koalas (*Phascolarctos cinereus*) [4]. We also present some red panda morphometric data to aid differentiation of these animals into age and sex classes.

Red pandas inhabit temperate forests with abundant bamboo in the Himalayan region between 2200 and 4500 m altitude [5,6,7,8,9]. This elusive species lives on mountains with moderate slopes and high canopy cover [5,7,10,11,12]. The species is cryptic and spends most of its time in trees only coming to ground to drink water, forage on bamboo shoots, and move from one site to another [13]. Red pandas are a solitary and territorial species, but males and females can be seen together during the mating season (January–March). The cubs are born in the monsoon season (June–August) and live with their mother until the next mating season [13]. These animals have reddish-brown fur on the dorsal side and blackish fur on the ventral side that helps them camouflage well in their natural habitat. Additionally, red pandas are shy and elusive. These attributes create challenges in spotting and capturing them at high altitude in remote mountainous terrain with a dense, complex pattern of vegetation comprising mixed-forest species, shrubs, vines, and bamboo.

Red pandas are endangered [9] and listed as an appendix I species in the Convention on International Trade in Endangered Species of Wild Fauna and Flora [14]. Their global population has declined by 50% in the last three generations [9]. Available studies have reported habitat loss and fragmentation as the major conservation challenges [15,16,17,18,19]. However, most of these studies are based on sign surveys, and they have not attempted to examine how this threatened species responds to habitat loss, fragmentation, and disturbances. Telemetry studies can provide much needed ecological information that can help advance conservation strategies to secure the survival of this threatened species. 

Studies have involved capturing and handling free-living red pandas in the past [13,20,21,22,23,24,25]. However, in those studies, the capture methods, both direct and indirect, are incompletely described [21,22,24,25]. Direct capturing methods employ climbing trees and capturing the red panda using the noose pole method [13]. Direct capturing has been employed in China using dogs to chase animals to a tree, but the method is poorly described [24,25]. Indirect methods include log traps [13,20,23], and leg-snare traps [23]. Red pandas have been trapped successfully in log traps designed for giant pandas, *Ailuropoda melanoleuca*, on two occasions in China [20,23]. A log trap is a type of box trap with wooden walls [26]. Of these, one red panda was caught as a by-catch in one of the traps targeted for giant pandas [20].

These methods appear to have some limitations and difficulties for use in the field. For instance, log traps that were reduced in size and baited with food and olfactory signals did not capture a single red panda in 427 trap days [13]. Despite being effective in animal trapping, leg-snare traps are not suitable for red panda due to predation risk [13]. Furthermore, leg snares can injure muscles and induce capture myopathy in trapped animals [1]. The direct trapping used by Yonzon [13] also raises questions about the welfare of study animals and field members’ safety due to possible falls from trees. To address these challenges, we developed a protocol for capturing and handling red pandas. This technique includes methods for red panda tracking, trapping, immobilization, handling, and release. Furthermore, recent publications about the red panda indicate that the tributaries of Brahmaputra river, the Yalu Zangbu and Siang rivers, act as a geographic barrier between the distributions of what are now recognized as two species [27,28]. These studies have highlighted that information on morphometric data of wild red pandas (*A. fulgens*) is limited to a single study [13]. Therefore, we also report morphometric data to enrich the natural history information of wild red pandas.

## 2. Materials and Methods

### 2.1. Study Area

The study was undertaken in the Ilam and Panchthar districts of eastern Nepal (27.10162° N, 87.98397° E) which borders Singalila National Park, India in the east. The area lies between 1500 and 3636 m elevation having temperate broad-leaf mixed and rhododendron forests. This area predominately has a sub-tropical and temperate climate with an average of 3500 mm annual rainfall [29]. Red pandas live in these forests, where there is an abundance of bamboo in the understory [7,11,12,13]. Annual temperatures of this area ranged from −1 to 28.9 °C with a mean temperature of 13.07 °C.

### 2.2. Tracking Red Pandas

**Figure 1:**
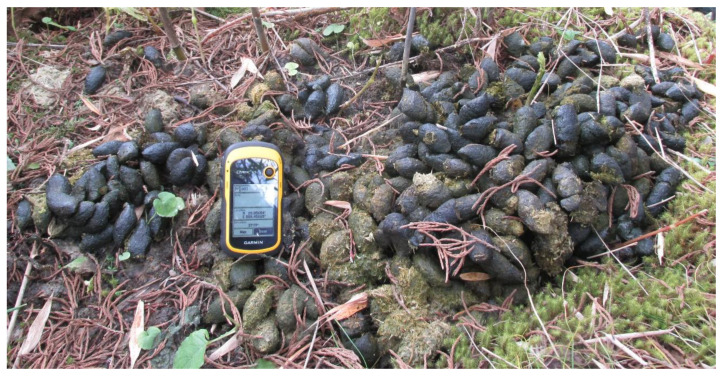
Red panda feces. Usually, fresh feces are greenish due to bamboo consumption in their diet. The color of the feces gradually fades with time. They use the same latrine sites for defecation where droppings of different ages can be easily seen (as in the figure). One animal can have many latrine sites within their home range. These latrine sites are believed to serve as territory markers. (Photo credit: Red Panda Network).

Red panda feces (Figure 1) are the most reliable evidence to track their presence in the wild [30]. These droppings are spindle-shaped, soft, moist, and light green, but coloration also depends on their diet and age [13,30]. Usually, they defecate 1–15 pellets in a single defecation and frequently visit latrine sites where more than 100 pellets can be found on tree branches, fallen logs, and the ground [5,10,13]. Red panda feces contain partially digested bamboo leaves and shoots [13,25]. Himalayan black bears (*Ursus thibetanus*) and Assamese monkeys (*Macaca assamensis*) in this region eat and fully digest bamboo shoots, but their feces are located on different substrates and are of different size and shape.

We tracked and captured red pandas from 21 September through 19 December 2019. We involved local people in red panda tracking and searched for direct sightings and fresh droppings. We had one snowfall event during this study. Red panda tracks are clearly visible on snow fields, so we also tracked them, following their tracks. Red pandas rarely flee if spotted in trees. When a red panda was located, we used two-way radio and mobile phones to summon other field crew while one person stayed at the base of the tree until all members arrived. The study area was accessible by jeep, but we had to access red panda encounter sites on foot carrying all necessary equipment.

### 2.3. Trap Design, Installation, and Trapping

**Figure 2:**
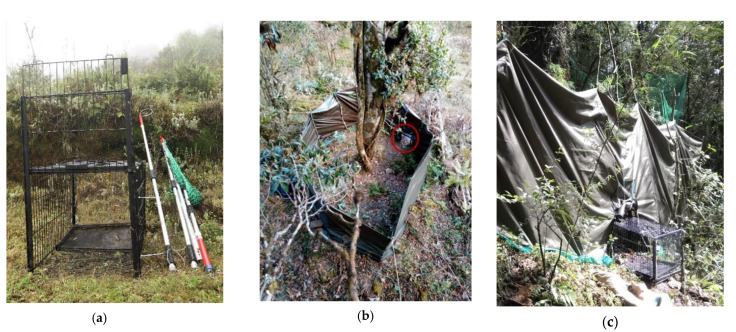
(**a**) Trapping instruments: cage trap, trapping poles, and net. (**b**) Fence built around a tree with a red panda. The fence was nearly 2.5 m high, built using a canvas sheet around the tree. Bamboo and wooden pegs were used to support the fence. This canvas sheet weighed nearly 25 kg. The cage trap (highlighted with a red circle) was placed downslope of the enclosure. (**c**) A view of the cage trap from outside the enclosure.

The trap used in our study was adapted from cage traps used for koalas in Australia [4]. With the help of a local metal workshop, we modified a dog cage made from an iron frame (80 × 48 × 50 cm). This collapsible cage trap weighed 13.5 kg. We modified the entrance and fitted it with an auto-closing shutter. The foot plate triggered the shutter to slide down and close as soon as the animal was inside the cage trap. We also replaced the frame of the opposite side with a transparent wall to appear as if it was an exit (Figure 2a).

Before placing the cage trap, we had to ensure that the treed animal did not flee. Connected tree branches provide access for red pandas to walk over the canopy to easily escape. Initially, each team member took a position under all possible trees from where the animal could climb down and escape. Then, we had to drive the red panda onto the most isolated tree having minimal connected branches with neighboring trees. Before we built the fence around the base of the tree (Figure 2b), we pruned connected branches and chopped off smaller saplings and shrubs around the base of the tree whilst minimizing disturbances to understory vegetation. Furthermore, our study area was managed by the local people as the community forest where the use of forest resources for timber, firewood, and fodder is permitted and commonly practiced.

Initially, we prepared a fence that was 1.5 m high [31]. However, a fence lower than 2 m failed to prevent red pandas from jumping over it in high slope areas. So we added another layer of green netting on top of the fence to increase its height if the site had a high slopes. We maintained the minimum height of the fence at 2.5 m above the ground in areas with steep slope, and the distance between the tree base and fence at between 2.5 and 4 m so that the animal could not jump over the fence. After the fence was completed, we installed the cage trap on the downhill side of the enclosure (Figure 2c). Finally, we placed a pair of trail cameras facing the cage trap from inside and outside the fence to record the movement and behavior of the trapped animal. 

Frequent visits by humans to trap sites may induce trap shyness in animals, which may affect trapping success [32]. Therefore, after setting the cage trap, we moved away and hid, disallowed the smoking of cigarettes, and waited until the animal climbed down and was caught in the cage trap. In three cases, the treed animal did not climb down within 3 h and we decided to intervene to minimize the stress. To do that, either one or two people climbed an adjacent tree and used a long bamboo pole with green leaves attached to the distal end to distract the animal by flapping it over the animal’s head in the same way as is done for koalas [33,34,35]. This approach worked well as we succeeded in less than half an hour, except in one case in which it took just over 2 h because this animal was in a particularly tall (22 m) and isolated tree.

**Figure 3:**
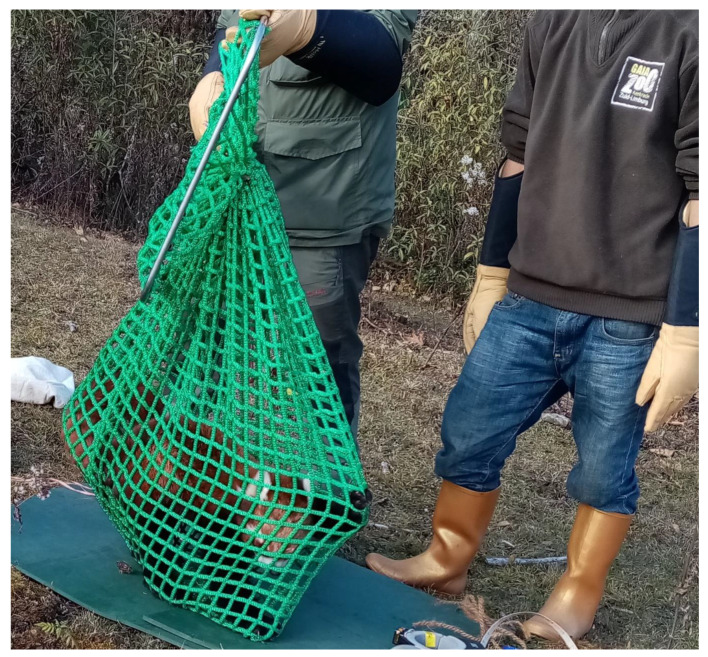
Net with a 1.5 m long handle was used to take the trapped animal out of the cage trap. The animal was taken out of this net after immobilization.

We covered the cage trap with hessian bags as soon as the panda was trapped to reduce their stress [2]. Three or four people went inside the fence and took the animal out from the trap with the help of a 1.5 m long net with a 50 × 50 cm mouth (Figure 3). Handlers had to wear protective gloves and masks to prevent biting, scratching and possible transmission of zoonotic diseases. Then, we tied the mouth of the net and weighed the animal using a handheld digital scale. We transferred the trapped animal to a pre-identified flat site close by for immobilization, collaring, and to record morphometric measurements and undertake a health examination.

We were also prepared 3.5 m extendable trapping poles if the animals were found on small trees with access from the ground. Owing to the risks to study animals and field crew, due to the difficult topography, we avoided trapping activities at night.

During the three months, we worked for 37 days, totaling 415 person-days and spent 3320 person-hours red panda tracking. On average, we deployed 11 (range 6–17) people/day.

### 2.4. Animal Handling and Immobilization

We followed guidelines developed by the American Society of Mammologists for animal handling [2]. To prevent possible transmission of rabies from red pandas, we vaccinated our team with the rabies vaccine. We administered drugs when the animal was inside the net. One person caught the red panda by the fur behind its neck and with the other hand held its rump, and then, it was placed on an insulated mattress. We planned to inject ketamine (6.6 mg/kg) and medetomidine (0.08 mg/kg) through intramuscular injection into a hind leg using a 20–22 G syringe [36]. We applied eye gel and closed the eyes of the red panda to protect them from dust, light, and other physical injuries [37]. The animal handling site had minimal staff to reduce noise for minimizing the stress to the animal.

After confirming immobilization by examining the eyes and responses to stimulus, we fitted a Global Positioning System (GPS) collar weighing between 224 and 229 g (LiteTrack Iridium 150 TRD, Havelock North, New Zealand). These collars had circumferences ranging from 210 to 230 mm, an auto drop off function, and battery life of one year. Auto drop off occurred at 60 weeks. Collars were set to provide one GPS fix every two hours and also had a Very High Frequency (VHF) transmitter. Based on trials with two red pandas in the Rotterdam zoo for six months, we fitted collars with the diameter of the index finger of an adult man between the neck and collar strap for adults, while for sub-adults, the gap was increased by 50%, which was within the range suggested by Dickinson et al. [38]. The collar battery and transmitter were placed onto the red panda ventrally and dorsally, respectively. Then, we recorded morphometric measurements and conducted a health examination after attaching the GPS collar. 

We differentiated male and female based on the relative size of mammary glands and the appearance of the genitals [31]. Since the trapping was conducted outside the birthing season, we categorized red pandas into only two age classes: adult and sub-adult. Animals with body weights ≥3.3 kg were considered adult and vice-versa [13]. The data recorded included body weight, body length (head to body), tail length, limb length and width, shoulder height, chest girth, and tail ring counts (Table S1). We also measured rectal temperature using a four-inch digital thermometer (MICROLIFE, Widnau, Switzerland). The average normal body temperature of the red panda is 99.7 °F [39]. We considered their temperature above 103 °F as hyperthermia and below 98 °F as hypothermia [40]. We calculated foot-load (gm/cm^2^) as the ratio of their body weight and the total area of their foot pads [41].

### 2.5. Revival and Release

We waited for 30 min to inject Atipamezole hydrochloride as an antidote into a hind limb after the administration of the last dose of anesthetic drugs; however, we had to administer it earlier in five cases when animals showed signs of recovery before 30 min.

We converted the cage trap into a recovery cage by padding the interior with the mattress and by covering it with a hessian bag. We waited for 30–60 min for the animal’s recovery. Then, we released the collared animal at the point of capture. None of the released animals climbed on the trapped tree, but GPS telemetry data showed that they did not avoid visiting the trapping site afterwards. A sedated animal could be easy prey for predators [2]. To ensure their safety, we followed and monitored the released animals at a distance of approximately 50 m for about 90 min until they regained normal movement. 

### 2.6. Data Analyses

We calculated mean (±SD) and median values of the morphometric measurements for parametric and non-parametric data, respectively. We have reported minimum and maximum value as a range for both the mean and the median. To test for significant differences between two groups—male and female, and adult and sub-adult—we employed two-sample t-tests if the data had a normal distribution with the same variance. Otherwise, we used a Wilcoxon rank sum test. We used the Shapiro–Wilk normality test to examine whether the data had normal or non-normal distribution. We used a Kruskal–Wallis rank sum test to examine the differences between the two groups with continuous variables. We considered *p* ≤ 0.05 as a threshold of statistically significant difference. We carried out all the statistical analyses in R [42].

## 3. Results

In total, we captured and collared 10 individual red pandas. The trapped animals comprised seven adults: three males and four females, and three sub-adults: two females and one male. The sub-adults were captured in two events 10 km apart. Of these, one male and one female were siblings. We assumed these sub-adults to be 6–7 months old, and they were accompanying their mothers when captured. We also collared their mothers. We made 33 sightings on 18 occasions and established a sighting rate as 0.89 pandas/day. We took 3.7 days to trap and collar one panda. It took nearly one hour to set up the cage trap.

We attempted trapping on 15 occasions but succeeded on nine attempts; thus, our trapping success rate was 0.6. We once captured two sub-adults in a single attempt. We found the trapping poles ineffective, as we did not catch any animals using them, although we tried on four occasions.

The mean ± SD (range) time taken to capture a red panda after spotting it was 136 ± 76 (50–317) min. We completed further processing, i.e., immobilization, collaring, recording morphometric measurements, and health examination in 38.5 ± 15.46 (21–70) min, and transferred animals into the recovery cage, where we kept them for 49.5 ± 12.44 (35–70) min before releasing. Anesthetic induction duration was 11.10 ± 9.86 (3–35) min and recovery duration was 28.3 ± 11.27 (15–50) min from the time the antidote was administered. We completed collaring in 2.75 ± 0.98 (2–4) min (Table S2). The mean body temperature of the collared animals was 103.1 ± 1.46 (100.7–104.9) °F. None of the collared animals were injured or died during this study. Based on GPS telemetry data and VHF tracking, all were known to be alive nine months after capture.

We trapped these red pandas from seven tree species: *Rhododendron arboreum* (*n* = 4), *Sorbus cuspidata* (*n* = 1), *Acer campbellii* (*n* = 1), *Ilex dipyrena* (*n* = 1), *Ilex fragilis* (*n* = 1), *Daphniphyllum himalayense* (*n* = 1), and *Magnolia cathcartii* (*n* = 1). Mean ± SD (range) height and diameter at breast height (dbh) of these trees were 15 ± 4.43 (7–22) m and 111 ± 101.2 (10.5–290), respectively. Mean tree cover and bamboo cover of these sites were 51.5 ± 18.17 (15–75)% and 43 ± 29.26 (0–80)%, respectively. All these sites were within 51 ± 55.4 (0–200) m of water sources. These animals were captured at a mean elevation of 2884 ± 59.6 (2810–3012) m.

The mean doses of ketamine and medetomidine used were 5.8 ± 0.71 mg/kg and 0.07 ± 0.02 mg/kg body weight, respectively. We administered atipamezole hydrochloride at 0.3 ± 0.14 mg/kg body weight, which is 5-fold more than the medetomidine dose.

The body weight (kg) of adults ($\bar{x}$ = 4.7, SD = 0.66) was nearly double that of sub-adults ($\bar{x}$ = 2.4, SD = 0.19, t_8_ = 5.28, *p* < 0.001, Table 1). The body weight of adult males did not vary from females (t_5_ = −1.65, *p* = 0.15). The adult’s body length (cm) was longer ($\bar{x}$ = 55.3, SD = 3.1) than that of sub-adults ($\bar{x}$ = 43.3, SD = 2.49, t_8_ = 5.8, *p* ≤ 0.001). Tail length (cm) followed a similar trend in adults ($\bar{x}$ = 43.7, SD = 2.43) and sub-adults ($\bar{x}$ = 36.3, SD = 2.62, t_8_ = 3.83, *p* ≤ 0.004). We observed nine and seven black rings on the tails of adults and sub-adults, respectively. We found no variation in the length and width of paws (analogous to human hands) between males and females, and adults and sub-adults (Table 1). Likewise, adult and sub-adult red pandas had no variation in the length and width of their pes (analogous to human feet, Table 1). However, mean male pes length (cm) was significantly longer ($\bar{x}$ = 13.5, SD = 0.94, t_5_ = −5.91, *p* ≤ 0.001) than that of female red pandas ($\bar{x}$ = 10.9, SD = 0.54). Pes width of males ($\bar{x}$ = 6.3, SD = 0.37) showed a similar trend, which was longer than that of females ($\bar{x}$ = 5.4, SD = 0.38, t_5_ = −3.1, *p* ≤ 0.026). 

Mean forelimb ($\bar{x}$ = 15.4, SD = 2.31) and hindlimb lengths ($\bar{x}$ = 12.8, SD = 0.5) were not different ($\chi_{4}^{2}$ = 3.22, *p* = 0.52, Kruskal–Wallis test). Similarly, we did not observe variation in the length of the forelimb and hindlimb between males and females, or adults and sub-adults. Adults had a greater shoulder height (t_5_ = 3.614, *p* ≤ 0.015) and larger chest girth (t_5_ = 5.3039, *p* ≤ 0.003) than sub-adults. We also found the adults with higher foot-load ($\bar{x}$ = 20.7, SD = 3.2) than that of sub-adults ($\bar{x}$ = 13.02, SD = 1.1, t_8_ = 3.59, *p* ≤ 0.007).

## 4. Discussion

Effective wildlife research and conservation often demands an animal in the hand to take samples [2,26], measurements [43,44], or to fit devices such as radio transmitters [2]. Capture and handling a wild vertebrate can be challenging [26,45] especially if the animal is cryptic [46,47,48], agile [46,48], or lives in difficult terrain [47]. Capturing wildlife can also be hazardous for the animal and the handler [2,46,49]. All these concerns apply to red pandas. However, after testing in the field, we found this fence-trap method to be an effective and relatively easy method for catching red pandas, which could be applied for other similar species with some modifications. It requires no expensive specialist equipment and could be applied in remote locations in developing countries.

The effort taken to track and capture red pandas in this study was much less than that of Yonzon [13], who spotted red pandas on 10 occasions and collared six individuals. We established 89.7 person-hours (0.89 panda/day) as our spotting rate, which is higher than previous reports [13]. There has been a community-based red panda conservation program in the study area for more than a decade, which also supports red panda tourism [50,51]. Thus, the involvement of experienced red panda trackers likely helped to increase the sighting rate in our study. Habituation to human presence due to increasing tourism activities could also have increased our sighting rate. Likewise, our success rate of trapping is similar to Yonzon [13]. Other studies [21,23,25] have not mentioned the research effort for the sighting and successful capture of red panda.

We had six unsuccessful trapping attempts where red pandas fled before we completed trap set up on three occasions, jumped over the fence on two occasions, and one individual escaped by jumping on another tree outside the fence. Jumping between trees has never been reported before [13]. However, no animals escaped after being caught in the cage trap. Yonzon [13] reported that two pandas escaped from his trap. This shows the effectiveness of our trapping method.

We observed red pandas on the ground on three occasions, while the balance of the sightings were in trees. When we attempted to catch a grounded animal, we had to approach them to make them climb a tree as has been done elsewhere [13,24,25]. In this study, we observed them fleeing some distance on the ground in front of the trackers before they climbed a tree. In China, dogs have also been used to chase red pandas to make them climb a tree [24], but this seems an unacceptably stressful way to capture red pandas.

Red pandas are solitary, elusive, and arboreal mammals living at low density in their natural habitat [13]. For this reason, non-directed trapping may take a very long time to make a successful catch. Such methods have been used in previous studies without success after even 427 trap days [13]. The failure could be due to the small-sized trap used. Our method was efficient in tracking and trapping in a short time with acceptable effort. This approach also possesses minimal risk to animal health and human safety. However, we experienced biting on two occasions while capturing and handling red pandas. Therefore, we suggest the use of protective gear such as gloves and boots to prevent biting and scratching as well as administration of the rabies vaccine to the entire field crew.

The anesthetic doses used in our study fall within the range recommended by Philippa and Ramsay [36]. We found new standard doses of ketamine (5.8 mg/kg) and medetomidine (0.07 mg/kg) as anesthetic agents and atipamezole (0.31 mg/kg) as an antidote for reversing the effect of medetomidine that were effectively used with red pandas. This dose also falls within the range suggested by Kreeger and Arnemo [37] except for ketamine. We had to top up ketamine in one animal that was moving; thus, we suspect that only a partial dose was injected. For this reason, the ketamine dose was high in that animal. However, the ketamine dose is less than previously used for immobilizing wild red pandas [13,21,22,24,25].

The average body temperature of red pandas was in the hyperthermia range, although the temperature was within the normal range in five individuals. The use of ketamine for immobilization induces hyperthermia in wild animals [40,52], which is further aggravated by stress [53]. This demonstrates the need for frequent body temperature measurements to be taken during animal handling [37,40]. Cold water enemas and an oxygen supply can be used to treat hyperthermia [40]. Exposure to the sun should be avoided during animal handling to minimize the risk of hyperthermia [37]. We had applied these measures except for cold water enemas to minimize the risk.

On average, collars were 6.19% of body weight, which was slightly higher than the “5% thumb rule” [54]. Even so, our method produced acceptable animal welfare outcomes, as all captured animals were known to be alive nine months after capture. Furthermore, three of the collared adult females successfully bred, and all three sub-adults dispersed and established their new range. Other studies on small mammals have used heavier devices up to 10% of their body weight [54]. Little evidence of negative effects of these heavier transmitters have been reported [55,56]. Furthermore, this 5% guideline was initially based on the maneuverability of flying vertebrates [57], which may need revisiting for small and medium-sized terrestrial vertebrates.

We found no significant variation in body weight and size between male and female red pandas. Similarly, we found sub-adults relatively smaller than adults, but it was not easy to differentiate them purely by sight. They look similar to adults when they are above six months old [13]. Only their tails provide a clue for distinguishing them from adults, as the tail length is comparatively shorter than that of adults. Consistent with the literature, we found no sexual dimorphism in size and coloration in red pandas [58,59].

This study extends the known range of body weights of free ranging males from 4.8 to 6.1 kg and females from 4.65 to 4.9 kg [13]. Our results also extend the tail length range from 44 to 49 cm for males and 38 to 43 cm for females [13]. However, we found the body length of adult males and females within the known range [13]. These weights and lengths are within the range of red panda in captive populations [58,60]. However, it is unclear whether those animals, used for measurements in captivity, were *A. fulgens* or *A. styani*. None of the animals were pregnant, as we deliberately captured them outside the mating and gestation period. Red pandas have high genetic variation across their distribution range [19,27], and such variation can change the phenotype of individuals [61]. Therefore, it is likely that future studies from other areas may extend these ranges in body size and weight.

Our morphometric data also serves as a baseline for the length and width of limbs, pes and paws, shoulder height, chest girth, and foot-load of wild red pandas, which have not been reported in any of the previous studies. Morphometric data available on the red panda from China [21,22,23,25] are of the Chinese red panda—*Ailurus styani* [27], which are not comparable with our study. Amongst three sub-adults, one was relatively heavier than the other two siblings, which may be due to the litter size effect as the weight decreases with increase in litter size [60]. The use of microchips in conjunction with GPS telemetry could allow long-term monitoring of the same individuals [62], which can provide information on movement, survival rate, longevity, growth rate, and many other biological and ecological aspects.

We found this trapping method efficient in catching and processing free-living red pandas. This method could be used for catching other arboreal species with some modification based on that animal’s size and behavior. To improve the efficiency of capturing, the diameter and height of the fence can be increased, but it will increase the weight of trapping equipment. We observed one individual jumping from 15 m high above the ground onto the bamboo canopy. Therefore, we suggest removing bamboo under the targeted tree canopy. However, the cut stumps should not be left open, as they can injure field crew and red pandas. Placing a net at 1–2 m high above the ground around the fence can save animals from injury and prevent escape. Maintaining silence on the trap site, avoiding smoking and not taking too many flash photos are equally important to minimize stress. Prolonged time taken for capturing and animal handling can be stressful for animals; it can also make animals flee. Involving more people in fence building could minimize the time required to construct the trap. Avoiding movement and maintaining silence after trap installation were important precautions to encourage the treed animal to climb down. This study provides new morphometric data that serve as a guide for field identification and provides baseline data for future studies on wild red pandas. More broadly, research is also needed to understand trapping-induced stress on wild red panda.

## 5. Conclusions

This research has demonstrated that it is possible to capture and handle arboreal, cryptic animals in a remote montane environment. We also demonstrated acceptable levels of animal welfare since all captured animals were known to be alive nine months after capture and some were breeding in the year after handling. Our results were no doubt enhanced by the knowledge and participation of local guides who take tourists to see wild red pandas in this area and are familiar with their habits. As a result of this research we were able to capture and GPS collar 10 animals and obtain information about them that was previously unreported. Capturing animals during the mating, gestation, and early cub-rearing seasons can affect their reproductive behavior and survival. Therefore, these critical times should be avoided for capturing animals.

All equipment was low cost and available locally. Hence, we believe that this method could be used, with modifications, for other species that might have hitherto been difficult to study. We recommend reporting trapping methods and morphometric measurements in detail in future studies.

## Figures and Tables

**Table 1:** Morphometric data of adult males (*n* = 3) and females (*n* = 4), and adults (*n* = 7) and sub-adults (*n* = 3). Central tendencies of variables are presented as mean and median for age and sex categories, and range is presented within parentheses as minimum and maximum values. Significant results are shown in bold.

Variables	Mean ± SD (Range)		Statistics *^1^	Mean ± SD (Range)		Statistics *^1^
	Adult	Sub-Adult		Male	Female	
Weight (kg)	**4.7 ± 0.66 (3.9–6.1)**	**2.4 ± 0.19 (2.3–2.7)**	**t_8_ = 5.28, *p* ≤ 0.001, *t*-test**	5.2 ± 0.9 (4.6–6.1)	4.4 ± 0.4 (3.9–4.9)	t_5_ = −1.65, *p* = 0.15, *t*-test
Body length (cm)	**55.2 ± 3.10 (50–60)**	**43.3 ± 2.49 (40–46)**	**t_8_ = 5.27, *p* ≤ 0.001, *t*-test**	56.3 ± 4.5 (54, 60)	54.5 ± 3.2 (50, 59)	t_5_ = −0.68, *p* = 0.52, *t*-test
Tail length (cm)	**43.7 ± 2.4 (41–49)**	**36.3 ± 2.6 (34–40)**	**t_8_ = 3.83, *p* ≤ 0.004, *t*-test**	45.6 ± 1.4 (43–49)	42.2 ± 0.8 (41–43)	t_5_ = −2.16, *p* = 0.08, *t*-test
Pes length (cm)	12 ± 1.4 (10–14)	11 ± 0.4 (10.5–11.5)	t_8_ = 1.09, *p* = 0.3, *t*-test	**13.5 ± 0.94 (13–14)**	**10.8 ± 0.54 (10–11.5)**	**t_5_ = −5.91, *p* ≤ 0.001, *t*-test**
Pes width (cm)	5.8 ± 0.6 (5–6.5)	5.3 ± 0.3 (5–5.6)	t_8_ = 1.19, *p* = 0.26, *t*-test	**6.3 ± 0.4 (6–6.5)**	**5.4 ± 0.38 (5–6)**	**t_5_ = −3.1, *p* ≤ 0.02, *t*-test**
Paw length (cm)	7.5 (7–11.5) *	7 (6–7) *	W = 6, *p* = 0.23, W-test	8 (7.5–11.5) *	7.35 (7–8) *	W = 2, *p* = 0.2, W-test
Paw width (cm)	5.5 (5–9) *	5 *	W = 9, *p* = 0.54, W-test	5.5 (5–9) *	5 (5–5.5) *	W = 4, *p* = 0.5, W-test
Forelimb length (cm)	15 (12–18) *	12.5 *	W = 18, *p* = 0.1052, W-test	16.1 ± 2.6 (13–18)	14.7 ± 2.2 (12–18)	t_5_ = −0.71, *p* ≤ 0.5, *t*-test
Hindlimb length (cm)	13 (12–13.5) *	11.5 (11.5–12) *	W = 18, *p* = 0.1008, W-test	12.8 ± 0.85 (12–13.5)	12.7 ± 1 (12–13.5)	t_5_ = −0.10, *p* = 0.92, t-test
Shoulder height (cm)	**27.5 ± 0.5 (27–28)**	**26.1 ± 0.24 (26–26.5)**	**t_8_ = 3.614, *p* ≤ 0.01, *t*-test**	28 ± 0.6	27	W = 0, *p* = 0.19, *t*-test
Chest girth (cm)	**34 ± 1.2 (33–36)**	**28.6 ± 0.94 (28–30)**	**t_8_ = 5.3039, *p* ≤ 0.003, *t*-test**	35 ± 1.7	33	t_5_ = −2, *p* = 0.18, *t*-test
Foot load (gm/cm^2^)	**20.6 ± 3.2 (15.7–24.1)**	**13 ± 1.1 (11.6–14.3)**	**t_8_ = 3.59, *p* ≤ 0.007, *t*-test**	18.2 ± 2.2 (15.7–19.5)	22.4 ± 2.8 (18.1–25.6)	t_5_ = 1.97, *p* = 0.10, *t*-test

* Medians; *^1^ two-sample *t*-test and Wilcoxon rank sum test are abbreviated as *t*-test and W-test, respectively.

## Data Availability

The data presented in this study are available in supplementary materials.

## Supplementary Materials

The following are available online at https://www.mdpi.com/2076-2615/11/4/921/s1, Table S1: Morphometric data of collared red pandas; Table S2: Details of anesthetics and time taken for capturing, immobilization and processing of captured animals.
