# Supplementary material for: Improved Trapping and Handling of an Arboreal, Montane Mammal: Red Panda Ailurus fulgens
Source: Animals (Basel). 2021 Mar 24;11(4):921. doi: 10.3390/ani11040921 (PMC8064068; doi:10.3390/ani11040921)
Supplement: Supplementary file 1 [file animals-11-00921-s001.pdf]

animals

Supplementary Materials

# Improved Trapping and Handling of an Arboreal, Montane Mammal: Red Panda *Ailurus fulgens*

**Damber Bista [1,*], Sonam Tashi Lama [2], Janno Weerman [3], Ang Phuri Sherpa [2], Purushotam Pandey [4], Madhuri Karki Thapa [5], Haribhadra Acharya [6], Nicholas J. Hudson [1], Greg S. Baxter [1] and Peter John Murray [7]**

1 School of Agriculture and Food Sciences, The University of Queensland, Gatton, QLD 4343, Australia; n.hudson@uq.edu.au (N.J.H.); gregbaxter36@gmail.com (G.S.B.)
2 Red Panda Network, Baluwatar, Kathmandu 44600, Nepal; sonam.lama@redpandanetwork.org (S.T.L.); ang.sherpa@redpandanetwork.org (A.P.S.)
3 Rotterdam Zoo, Blijdorplaan 8, 3041 JG Rotterdam, The Netherlands; j.weerman@diergaardeblijdorp.nl
4 Directorate of Livestock and Fisheries Development, Province no.1, Biratnagar 56613, Nepal; wildelephant.pandey@gmail.com
5 Department of Forest and Soil Conservation, Babarmahal, Kathmandu 44600, Nepal; madhureethapa@gmail.com
6 Department of National Parks and Wildlife Conservation, Babarmahal, Kathmandu 44600, Nepal; hbacharya07@gmail.com
7 School of Sciences, University of Southern Queensland, West St, Darling Heights, QLD 4350, Australia; peter.murray2@usq.edu.au
* Correspondence: d.bista@uqconnect.edu.au or damb.2007@gmail.com

**Citation:** Bista, D.; Lama, S.T.; Weerman, J.; Sherpa, A.P.; Pandey, P.; Thapa, M.K.; Acharya, H.; Hudson, N.J.; Baxter, G.S.; Murray, P.J. Improved Trapping and Handling of an Arboreal, Montane Mammal: Red Panda *Ailurus fulgens*. *Animals* **2021**, *11*, x. https://doi.org/10.3390/ani11040921

Academic Editor: Andrew W. Claridge

Received: 01 March 2021
Accepted: 20 March 2021
Published: 24 March 2021

**Publisher's Note:** MDPI stays neutral with regard to jurisdictional claims in published maps and institu-tional affiliations.

**Copyright:** © 2021 by the authors. Submitted for possible open access publication under the terms and conditions of the Creative Commons Attribution (CC BY) license (http://creativecommons.org/licenses/by/4.0/).

**Simple Summary:** Capture and handling is essential to study some biological and ecological properties of free-ranging animals. However, capturing an arboreal and cryptic species such as the red panda is challenging due to the difficult terrain, their elusive nature, and potential risks to human and animal safety. We developed and successfully tested a protocol for tracking, capture, immobilization, and handling of red pandas. This method could also be used, with some modifications, for other arboreal species. This study extends the known range of body weight and length of free-ranging red pandas. We also report some new morphometric data that could serve as a guide for field identification.

**Table S1.** Morphometric data of collared red pandas.

| Animal | Sex [1] | Age [2] | Weight (kg) | Length (cm) | | | | Number of black rings[3] | Paw (cm) | | Pes (cm) | | Temperature (°F) |
|---|---|---|---|---|---|---|---|---|---|---|---|---|---|
| | | | | Body | Tail | Forelimb | Hindlimb | | Length | Width | Length | Width | |
| Paaru | F | A | 4.6 | 59 | 41 | 14 | 14 | 9 | 7 | 5 | 11 | 5 | 102.4 |
| Dolma | F | A | 3.9 | 55 | 42 | 12 | 10 | 9 | 7.2 | 5 | 10 | 5.2 | 105.1 |
| Chintapukamal | M | A | 6.1 | 60 | 49 | 17.5 | 13.5 | 9 | 11.5 | 9 | 14 | 6.5 | 104.9 |
| Mechhachha | F | S | 2.7 | 44 | 40 | 12.5 | 11.5 | 7 | 6 | 5 | 11.5 | 5.6 | 100.7 |
| Senehaang | M | S | 2.3 | 40 | 35 | 12.5 | 12 | 7 | 7 | 5 | 11 | 5.5 | 101.8 |
| Bhumo | F | S | 2.3 | 46 | 34 | 12.5 | 11.5 | 7 | 7 | 5 | 10.5 | 5 | 101.4 |
| Ngima | M | A | 4.6 | 54 | 45 | 13 | 13 | 9 | 8 | 5 | 13 | 6 | 104.5 |
| Brian | M | A | 4.8 | 55 | 43 | 18 | 12 | 9 | 7.5 | 5.5 | 13.5 | 6.5 | 104.9 |
| Ninaammaa | F | A | 4.1 | 50 | 43 | 18 | 13.5 | 9 | 8 | 5.5 | 11.5 | 6 | 102.7 |
| Prahladevi | F | A | 4.9 | 54 | 43 | 15 | 11.5 | 9 | 7.5 | 5.5 | 11 | 5.5 | 103.8 |

[1] Male (M) and Female (F) [2] Adult (A) and Sub-adult (S) [3] Tail has alternating black and red rings.

**Table S2.** Details of anesthetics and time taken for capturing, immobilization and processing of captured animals.

| Animal | Drugs (mg) | | | Time Taken (min) | | | | | |
|---|---|---|---|---|---|---|---|---|---|
| | Ketamine | Medetomidine | Antisedan | Induction of first effect | Capturing | Immobilization | Collaring | Processing | Recovery |
| Paaru | 50 | 2 | 0.25 | 4 | 143 | 35 | 4 | 57 | 35 |
| Dolma | 26.5 | 0.53 | | 5 | 317 | 7 | 2.5 | 21 | 56 |
| Chintapukamal | 32.5 | 0.35 | 3.5 | 2 | 90 | 6 | 5 | 53 | 49 |
| Mechhachha | 17.5 | 0.2 | 1 | 3 | 63 | 9 | 2 | 32 | 45 |
| Senehaang | 15 | 0.15 | 0.75 | 5 | 185 | 25 | 2 | 38 | 36 |
| Bhumo | 15 | 0.15 | 0.75 | 3 | 185 | 6 | 2 | 70 | 54 |
| Ngima | 25 | 0.3 | 1.5 | 2 | 145 | 3 | 2 | 26 | 45 |
| Brian | 25 | 0.3 | 1.5 | 4 | 50 | 8 | 3 | 35 | 70 |
| Ninaammaa | 20 | 0.2 | 1 | 6 | 75 | 8 | 2 | 22 | 70 |
| Prahladevi | 25 | 0.3 | 1.5 | 2 | 104 | 4 | 3 | 31 | 35 |
